# Assessing Probabilistic Risk Assessment Approaches for Insect Biological Control Introductions

**DOI:** 10.3390/insects8030067

**Published:** 2017-07-07

**Authors:** Leyla V. Kaufman, Mark G. Wright

**Affiliations:** Department of Plant and Environmental Protection Sciences, University of Hawaii at Manoa, 3050 Maile Way, Honolulu, HI 96822, USA; leyla@hawaii.edu

**Keywords:** risk assessment, origin, natural enemies, non-target species

## Abstract

The introduction of biological control agents to new environments requires host specificity tests to estimate potential non-target impacts of a prospective agent. Currently, the approach is conservative, and is based on physiological host ranges determined under captive rearing conditions, without consideration for ecological factors that may influence realized host range. We use historical data and current field data from introduced parasitoids that attack an endemic Lepidoptera species in Hawaii to validate a probabilistic risk assessment (PRA) procedure for non-target impacts. We use data on known host range and habitat use in the place of origin of the parasitoids to determine whether contemporary levels of non-target parasitism could have been predicted using PRA. Our results show that reasonable predictions of potential non-target impacts may be made if comprehensive data are available from places of origin of biological control agents, but scant data produce poor predictions. Using apparent mortality data rather than marginal attack rate estimates in PRA resulted in over-estimates of predicted non-target impact. Incorporating ecological data into PRA models improved the predictive power of the risk assessments.

## 1. Introduction

The rate of biological invasions globally has increased dramatically in the past 500 years due to an increase in human activities such as transportation, migration, and commerce [1]. Invasive species cause direct and indirect effects on organisms living in the environment they invade, and therefore threaten biodiversity, agriculture, and human health. Besides the environmental impacts, invasive species cause major economic losses in different sectors of the U.S. economy [2]. The practice of classical biological control (CBC), as the intentional transfer of natural enemies from one place to another, has traditionally been used as a tool to fight invasive species in agricultural settings and is now also being used to control invasive species in natural areas [3,4].

The enemy release hypothesis states that organisms become invasive in a new area because they have escaped the natural enemies that suppress their populations in their area of origin. Exotic species thus have an advantage over competitors in areas of introduction where indigenous species are still suppressed by their indigenous natural enemies [5]. Therefore, CBC works under the premise that the reestablishment of top-down control by introduction of natural enemies will reduce the populations of invasive species and therefore restore balance [3].

The history of biological control provides many examples of remarkable successes [6]. Prime examples include the introduction of the Australian lady beetle *Rodolia cardinalis* (Mulsant) (Coleoptera: Coccinellidae) to control the cottony cushion scale, *Icerya purchasi* Maskell, an introduction that is credited with saving the California citrus industry, and the introduction of *Anagyrus lopezi* (DeSantis) (Hymenoptera: Encyrtidae) from South America to control the cassava mealybug *Phenacoccus manihoti* Matile-Ferrero, in Africa, recognized to have saved many people from starvation. Those are just two of many other remarkable examples; Van Driesche et al. [7] provide a comprehensive review of the benefits of biological control of pests. Besides the economic benefits of this practice, the use of biological control has also led to a reduction in the use and dependence on pesticides, with coincidental benefits in terms of human and environmental health.

The potential and realized positive effects of biological control have been recognized for over a century. Many practitioners have long considered this practice environmentally safe, benign, risk-free, and a natural phenomenon [8,9,10,11]. Even though awareness of potential negative effects was also expressed over a century ago [12], it was only in the 1980s that classical biological control was first severely criticized as posing serious environmental risks [13,14]. At the center of this criticism was the issue of host specificity, or the lack thereof [15]. Biological control agents were implicated in the reduction of populations of native and desirable species, and were blamed in some cases to be agents of extinction [13,14]. Soon after, regulators and researchers were calling for more rigorous screening methods in the USA [15] and revisiting means of predicting positive or negative impacts of biocontrol agents. Some authors went so far as to call for the cessation of biological control [16].

Throughout the history of biological control, the most adverse effects in terms of non-target attacks have been due to the release of highly polyphagous species [17,18]. Generalist biological control agents were at one point considered superior not only because they could potentially control several pests but they could also persist on native insects at times when the target pest was rare. Such releases of generalist species were made before the concerns about environmental impacts were appreciated. In the specific case of Hawaii, after concerns were raised the state experienced an overall reduction of biological control introductions due to the implementation of rigid regulations [19].

At the time the potential for environmental impacts of introduced biological control agents was recognized in the USA, the need for regulations and guidelines for introductions as well as for comprehensive risk assessment (RA) frameworks became apparent.

Several protocols have been developed for the selection of non-target species for screening and host range determination [20,21,22,23,24,25]. Some countries such as New Zealand, Australia and South Africa as well as countries within the European Union have developed their own regulations and RA frameworks. For the most part they have similar criteria, but they involve different procedures and work under different guidelines.

The United States has no comprehensive RA methodology adopted for insect biological control introductions, and this situation is further complicated by a range of regulations at the state level. Hawaii represents a unique case, with a long history of biological control introductions, and has been the center of controversy regarding non-target effects. This has resulted in overly-restrictive regulations being implemented there [19]. The state of Hawaii and the USA in general should greatly benefit from the adoption of a general quantitative RA framework to characterize the potential risks of proposed biological control introductions.

**Figure 1:**
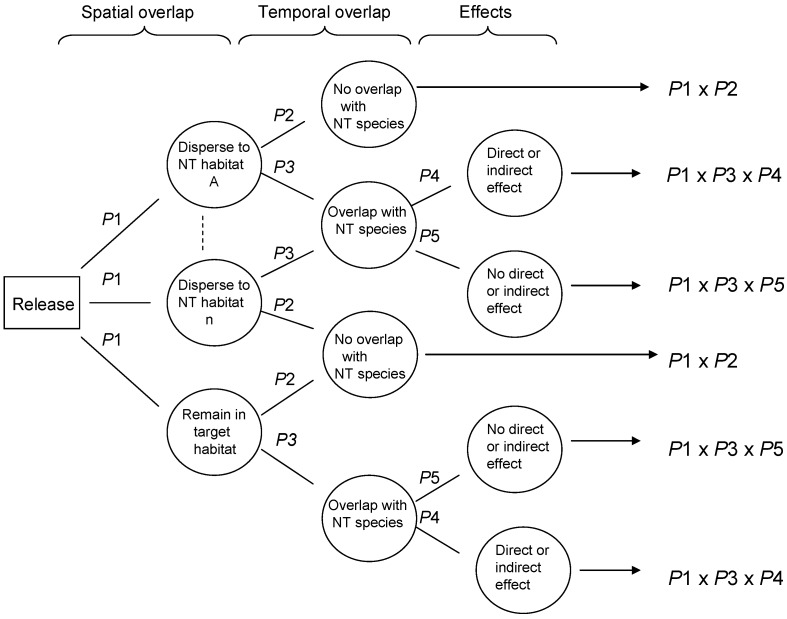
Conceptual precision tree. Circles contain contingencies (spatial overlap, temporal overlap, and effects); the probability of each occurring is given in connecting lines. The overall probability of each outcome is estimated by multiplying the *p* values along each branch.

Risk assessment in the field of biological control evaluates the likelihood that adverse ecological effects may occur, and the expected magnitude of any adverse effects, as a result of a release of a purposely introduced biological control agent. Two general RA frameworks have been proposed with great potential to be widely adopted. van Lenteren et al. [24] proposed a semi-quantitative environmental RA approach for inundative biological control agents, which was later improved and expanded to address classical biological control agents, in a stepwise procedure that identifies biological control agents with high potential non-target risk early in the process, therefore avoiding unnecessary research and use of resources [25,26]. Wright et al. [27] proposed a probabilistic RA (PRA) approach for either classical or inundative biocontrol agents, using conditional probabilities in a Bayesian approach to estimating risk, illustrated conceptually in Figure 1.

It has already been shown that the risk posed by biological control agents can vary spatially and temporally within the area of introduction [28,29,30,31,32]. Nevertheless, current RA procedures lack comprehensive incorporation of spatial and temporal components to characterize the risk. In attempting to predict how a biological control agent will respond to potential new hosts in a new area of introduction, it is important to elucidate its behavior in the area of origin and other areas of distribution to predict the nature of potential relationships with target species and potential non-target species.

The aim of this paper is to validate the probabilistic risk assessment methodology proposed by Wright et al. [27] using retrospective and current data from a study system in Hawaii. Wright et al. [27] proposed that non-target risk could be assessed using decision trees, which estimate the conditional probabilities of certain dependent events occurring, in this case non-target exploitation. The proposed approach included probabilities associated with various parasitoid behaviors, to conduct risk assessment for biological control agents, incorporating host range, ecological and behavioral aspects of parasitoid ecology. The probability of parasitism occurring at various levels may be modeled and incorporated into assessments that predict the ‘worst case’ (high probability of high parasitism) vs. the ‘best case’ (low probability of any non-target parasitism), through constructing probability distributions for each ecological aspect, using field collected data. The validation process addresses the three questions below using data from recently published work addressing the impacts of purposefully introduced and accidentally introduced parasitoids on an endemic Hawaiian moth, *Udea stellata* (Crambidae) [33,34,35,36]. *Udea stellata* is a moderately abundant species specific to an endemic plant, which occurs across a fairly broad ecological gradient in Hawaii, under a range of environmental conditions. It is considered to be a significant source of food for certain Hawaiian birds. We selected *U. stellata* as a study species as a representative of a typical Hawaiian insect, rather than one that was threatened, so that the impacts of introduced insects could be assessed, rather than other impacts such as habitat loss. Citations 33–36 report the results of studies on parasitoids attacking *U. stellata* in Hawaii, quantifying the parasitoid assemblage, levels of mortality (both apparent mortality, measuring proportion of hosts parasitized by a parasitoid in random samples; and marginal mortality, estimated generational mortality inflicted by a parasitoid, in the presence of other mortality factors), and ecological correlates of parasitoid distributions.

Key questions we addressed were: Would it have been possible to predict that *Cotesia marginiventris* (Braconidae), *Meteorus laphygmae* (Braconidae), and *Trathala flavoorbitalis* (Ichneumonidae) would attack *U. stellata* based on earlier published data, and would it have been possible to predict their level of impact on this non-target species using PRA? How useful are estimates of marginal vs. apparent mortality (and measures of uncertainty in both) in conducting PRA? Are there any key ecological variables that would be worth considering in the hypothetical case that any of the species addressed will be considered for introduction in another place?

### 1.1. Validation of the Probabilistic Risk Assessment Methodology

This section presents the procedures used to answer the four main questions stated in the introduction, regarding the validation of a PRA approach applied to biological control agents. A summary overview of risk assessment methods is provided in Supplementary Materials.

Question 1: Would it have been possible to predict that any of the three introduced parasitoid species would attack *U. stellata* based on published historical data, and would it have been possible to predict their level of impact on this non-target species using data gleaned from the published literature using PRA?

To address the first part of the question, whether older published records would have given an indication that any of the three introduced parasitoid species would attack *U. stellata*, reports on parasitism by *C. marginiventris*, *M. laphygmae*, and *T. flavoorbitalis* were sought in older published records (from 1913 to date of introduction or first record) by consulting printed copies of the abstract journal *Review of Applied Entomology*. The Thompson Catalogues of Host-parasitoid Associations [37,38] were also consulted (similar procedure described by [39,40]).

The second part of the question, whether it would have been possible to predict their level of impact on *U. stellata* based on historical data using PRA, validates a probabilistic risk assessment approach [27] with precision trees. As far as possible, probabilistic methods were applied by deriving probabilities of parasitism under different circumstances, from available data that could be gathered on the biology and ecology of the species being examined, from the literature as if it would have been available prior to the ‘introduction’ of the species of interest. These data form the basis for predictions (hypotheses) available at the ‘time of introduction’ and provide the first step for the validation. As actual non-target impacts were measured during the course of a number of studies supporting this work [33,34,35,36], it should be possible to corroborate the probabilistic model “predictions” with contemporary quantitative field data (presented as probability distributions). The validation was carried out by developing precision trees based on published data from earlier studies, compared to current actual impacts measured in the field.

The parasitoid species used for the validation section were *C. marginiventris, M. laphygmae*, and *T. flavoorbitalis,* with *U. stellata* as the non-target organism. Validation was not performed for the other species that parasitize *U. stellata*, *Diadegma blackburnii*, *Pristomerus hawaiiensis*, *Casinaria infesta* and *Triclistus* nr. *Aitkeni*, since little information was available in the literature on the latter four.

### 1.2. Problem Formulation

#### 1.2.1. The Non-Target Host *Udea stellata* (Butler)

The genus *Udea* (Lepidoptera: Crambidae) is a very large group that occurs in the Americas, Eurasia, and into the Pacific [41]. Hawaii has 44 endemic species in this genus [42]. *Udea stellata* was first described by Butler in 1883. *Udea stellata* (Lepidoptera: Crambidae) is a multivoltine species that undergoes six larval stages. The larvae feed on endemic host plants in the genus *Pipturus* (Urticacea, common name “mamaki”). All six larval stages are susceptible to parasitism by at least one of seven introduced parasitoid species associated with them [35].

#### 1.2.2. Habitats Supporting the Non-Target Species, *U. stellata*

The endemic host plants of *U. stellata*, *Pipturus* spp. are typically found in mesic forests. They occur across a moderate elevation gradient (from sea level to 1400 m). Most *Pipturus* populations in Hawaii currently occur in habitats with some level of disturbance by alien plant species. For this study, *Pipturus* habitats in Hawaii were classified based on landuse/landcover (LULC, State of Hawaii Office of Planning) and fall into two categories: shrub and rangeland; and evergreen forests. Even though *Pipturus* spp. do not occur in grassland habitats per se, they do occur in shrubland areas at the margins of grasslands. Elevation was also used to classify habitats where *Pipturus* spp. occur, since it is one of the ecological factors found to significantly influence the parasitoid assemblage associated with *U. stellata* [36]. Non-target habitats below 500 m were considered low elevation, habitats between 500 and 900 m were considered medium elevation, and habitats above 900 m were considered high elevation.

#### 1.2.3. Introduced Parasitoids Exploiting *U. stellata*

*Cotesia marginiventris* (Cresson) (Hymenoptera: Braconidae): a solitary endoparasitoid native to the West Indies [43]. Boling and Pitre [44] studied the biology of this species in the hosts *Tricoplusia ni* (Hübner), *Pseudoplusia includes* (Walker), and *Heliothis virescens* (Fabricius). *Cotesia marginiventris* undergoes three larval stages. Soon after molting to the third instar, the parasitoid larva exits its host to spin a white cocoon and pupates inside it. The host dies shortly after parasitoid emergence. Females have short ovipositors and have a preference to oviposit in early instar larvae.

*Cotesia marginiventris* has been purposely introduced to Cape Verde (Africa), Karnakata (India), Trinidad and Tobago (Caribbean), Australia (Oceania), and Hawaii (North America). Fred Bianchi, assistant entomologist of the Hawaiian Sugar Planters’ Association (H.S.P.A) Experimental Station, introduced this parasitoid to Hawaii from Brownsville, Texas to control the sugar cane pest *Spodoptera exempta* [45,46,47]. The introduction was made during the last half of 1942. A total of 4277 adults were distributed to Kauai, Oahu, Maui, and Hawaii. It became quickly established in the islands. At the time of the introduction there was no concern about possible non-target effects. Funasaki et al. [48] and Henneman and Memmott [49] report this species attacking other exotic species as well as native insects in Hawaii (Table 1). On the non-target host *U. stellate, C. marginiventris* can parasitize second, third, and fourth instars and emerges from the host when it is either fifth or sixth instar [35].

*Meteorus laphygmae* (Viereck) (Hymenoptera: Braconidae) is a nocturnal solitary koinobiont endoparasitoid [50,51]. Fernández and Terán [50] studied the biology of *M. laphygmae* in the host *Spodoptera frugiperda* (J. E. Smith). *Meteorus laphygmae* undergoes three larval stages; the first two develop inside the host and soon after molting to the third instar the larva emerges from the host to immediately start spinning a cocoon, inside which it metamorphoses to the pupal stage. The pupal cocoon is suspended from a thread anchored on foliage, as a protection from natural enemies [45].

This parasitoid is currently distributed in many states in the continental United States as well as Hawaii, Mexico, Central America (Trinidad and Tobago), and South America (Colombia and Venezuela) [52]. This parasitoid was purposely introduced to Hawaii by Bianchi, from Brownsville, Texas, to control the sugarcane pest *S. exempta*. The introduction process was done from June to November of 1942, and resulted in establishment of the species. A total of 3900 wasps were distributed on Oahu, Maui, Kauai, and Hawaii [45,47].

Funasaki et al. [48] and Henneman and Memmott [49] reported *M. laphygmae* parasitizing other exotic species as well as non-target native species in Hawaii (Table 2). *M. laphygmae* can parasitize second to fifth instar larvae of *U. stellate,* and emerges from the host’s sixth instar [35].

*Trathala flavoorbitalis* (Cameron) (Hymenoptera: Ichneumonidae) is a solitary, parthenogenetic endoparasitoid [35]. The literature reports that this parasitoid is a larval-pupal parasitoid [53] but it was never observed emerging from a pupa of *U. stellata* in Hawaii [35].

*Trathala flavoorbitalis* occurs in Asia (China, India, Indonesia, Japan, Myanmar, Philippines, Singapore, and Sri Lanka), Europe (Russian Federation), Hawaii, and Australia. This parasitoid has not been purposely introduced anywhere in the world. It is considered an important indigenous biological control agent of many Lepidopteran pests in Asia [36]. *Trathala flavoorbitalisis*, an adventive species to Hawaii, was first detected in 1910 [54] but for the purpose of this risk assessment validation is considered intentionally introduced for the sake of risk modeling. This species has been reared from many adventive and endemic species in Hawaii (Table 3). *T. flavoorbitalis* can parasitize first to fifth instar larvae of *U. stellata*, and emerges from the host at the sixth instar [35]. 

### 1.3. Analysis

For the purpose of the validation, *U. stellata* is considered to be the only non-target species of concern for each of the three parasitoids. In order to explore ways to express uncertainty created by the limited available data, two different scenarios were modeled: the average outcome, and the worst-case scenario. Average-outcome scenarios were modeled based strictly on the information found in the literature, whereas the worst-case scenario was based on the assumption that the published data were not comprehensive, and that hypothetically the agents could successfully overlap with *U. stellata* geographically, and locate and parasitize susceptible stages of *U. stellata* at similar rates as the target host.

### 1.4. Exposure Assessment

Published reports on parasitism were gathered for the three different parasitoids. Information on elevation and habitat type (land use and land cover: LULC) reported in the literature was used to predict potential spatial overlap with *U. stellata*. In the risk models, the best-case outcome scenario for the three parasitoids assumed no spatial overlap (probability, *p* = 0) with habitats that maintain populations of *U. stellata* (evergreen forests and scrubland/shrubland sites) at low (below 500 m), medium (between 500 and 900 m) and high elevation (above 900 m) sites, based on the fact that the published data only report parasitism in agricultural and grassland areas in the provenance of the parasitoids [56,57,58,59]. The modeled worst-case scenario assumed that published data were biased by habitat type and sampling, and that the three parasitoids could occur in other habitats such as forest and shrub land areas at the three elevation ranges, and therefore overlap spatially with *U. stellata* populations (*p* = 1.0).

Temporal overlap: Larvae of the non-target species, *U. stellata,* are perennially present [33]. Because of the seasonally equable environmental conditions in Hawaii, it was assumed that the three parasitoids are also perennially active. Therefore full synchronization with susceptible stages of *U. stellata* is considered to be the most likely scenario for all three cases (probability of temporal overlap, *p* = 1.0).

The effects assessment phase of the PRA was based on published records of parasitism for each of the three parasitoid species from outside of Hawaii. Parasitism data obtained from the literature were used to construct probability distributions for each elevation level (low, medium, and high). 

The average outcome scenario assumed that each of the parasitoid species would attack only hosts in the families with records of parasitism obtained from the published data. The worst-case scenario assumed that potential hosts in the family Crambidae (specifically *U. stellata*) will also be subject to parasitism.

For *C. marginiventris* and *M. laphygmae*, parasitism data were obtained for the target hosts *S. frugiperda* and *S. exigua* at at low, medium, and high elevations [56,58] in Mexico (sites at similar latitude to Hawaii). For *T. flavoorbitalis*, parasitism rates were available for the target pest *Antigastra cautalaunalis* in India only at low-elevation sites (below 500 m) [57,59] (latitude: 29 and 23 degrees north, respectively).

### 1.5. Risk Characterization

**Figure 2:**
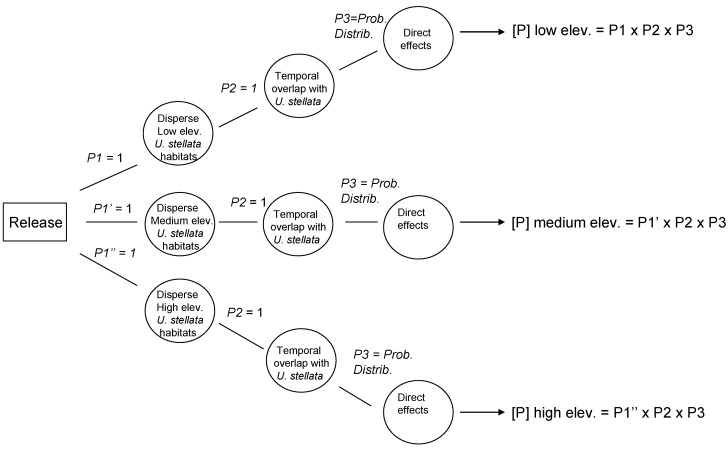
General precision tree for worst-case scenario outcomes in terms of non-target impact in Hawaii. *P*1 represents the probability of spatial overlap, *P*2 represents the probability of temporal overlap, and *P*3 represents the probability distribution of all possible outcomes for parasitism (effects on a non-target species). P1, P1’ and P2” refer to *p*-values associated with each initial dispersal option.

Data from the exposure assessment were used to construct overall probability distributions of parasitism rates for each of the given scenarios, and these data were used in the development of the precision-trees (Figure 1). Figure 2 shows the precision tree for the worst-case scenario.

Apparent mortality data [35] were used to construct probability distributions and validate the results obtained from the precision trees (based on information obtained from previously published data). 

Uncertainty analyses (Monte Carlo, MC, simulations) were conducted using Crystal Ball^®^ software [60] and each simulation was run with 2000 trials. For validation purposes, probability distributions were also generated for the observed parasitism rates in Hawaii, using recently published data [33,34,35,36]. The software presents results of the MC simulations graphically as probability/frequency distributions of all possible outcomes. Data generated during the simulations were extracted and analyzed using nonparametric statistics. The Mann–Whitney Rank Sum Test was used to test for significant differences between data when we had only two groups, and the Kruskal–Wallis ANOVA on ranks was used when we had data for multiple groups. The statistical analyses were done using SigmaStat 3.1 (Systat Software, San Jose CA, USA). 

Question 3: How different are the outcomes using estimates of marginal attack rates versus apparent mortality (and measures of uncertainty in both) in conducting probabilistic risk assessment?

Apparent mortality rates (field parasitism, [35]), Table 4) and *U. stellata* marginal attack rates (from life table studies of cohorts of larvae, [34], Table 5) were used for this analysis for each of the three parasitoid species to build probability distributions. Data from the life table studies reported in [34] and from field surveys of apparent mortality ([35], Table 4) were pooled across exposure trials and surveys, respectively, for the development of probability distributions. Probability distributions of marginal attack rate and apparent mortality were used as input variables in the probabilistic models (run in Crystal Ball^®^) keeping all other variables of the model constant (spatial and temporal overlap), to run the simulations. This provided estimates of mean mortality in the risk-analyses using measures of apparent or marginal mortality. Data generated during the simulations were extracted, and the Mann–Whitney Rank Sum Test was used to test for significant differences between the two groups.

Question 4: Are there any key ecological variables that would be worth considering in the hypothetical case that any of the three parasitoid species might be considered for introduction to another location or are generally worth considering in any risk assessment?

The results of the comparison of the prediction model and actual field parasitism in Hawaii (Question 1) were used to indicate which aspects of a potential biological control agent’s biology and ecology need to be investigated to provide a robust estimate of their non-target potential.

## 2. Results and Discussion

### 2.1. Question 1: Would It Have Been Possible to Predict that Any of the Three Parasitoid Species Would Attack U. stellata Based on Published Historical Data, and Would It Have Been Possible to Predict Their Level of Impact on this Non-Target Species Using PRA?

#### 2.1.1. *Cotesia marginiventris*

Table 6 presents the list of known hosts in the native range of *C. marginiventris*, mostly Noctidae, known before this species was introduced to Hawaii in 1942. The published literature also reports parasitism on species in the family Sphingidae and Crambidae. Within the family Crambidae, *C. marginiventris* was known to parasitize *Udea rubigalis* (Table 6), a species native to continental North America and a pest of celery, also known as the celery leaf-tier. Other species in that genus, such as *U. stellata*, could thus have been predicted to be potential non-target hosts (Hawaii has 44 described endemic representatives in this genus). Records following introduction in Hawaii show that besides attacking *U. stellata*, *C. marginiventris* also parasitized the other endemic non-target hosts in the families Crambidae, Tortricidae, Geometridae, Oecophoridae, and Noctuidae (Table 1).

**Figure 3:**
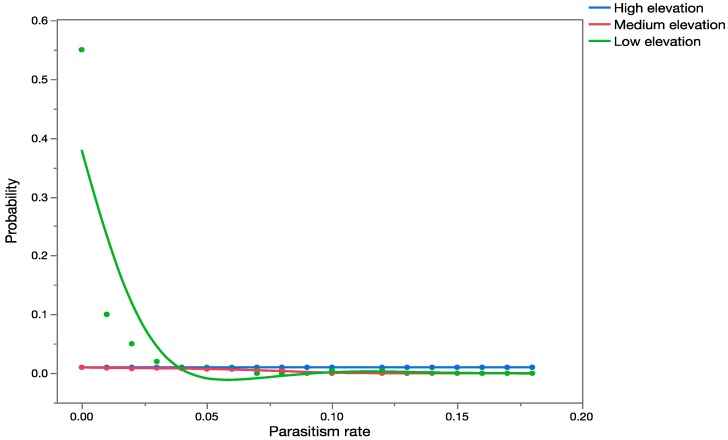
Overlay chart showing forecast of expected parasitism rates (proportion on *x*-axis) for *C. marginiventris* at low-, medium-, and high-elevation sites using early published data (Table 6).

Based on the literature, it was also predicted that *C. marginiventris* would only occur in agricultural and grassland areas. Figure 3 shows the results of the PRA based on those data. The data used were from an extensive field survey for parasitoids associated with *Spodoptera* in Mexico (sites at similar latitude to Hawaii). Results of the PRA are presented in graphs, which are probability/frequency distributions of all possible parasitism rates (expressed as proportions on the *x*-axis) based on records of parasitism. They represent the worst-case scenario, which assumes spatial overlap and temporal overlap (*p* = 1 for both factors) with populations of *U. stellata,* and that parasitism rates on *U. stellata* will be similar to those recorded in the historical data for its target hosts, *Spodoptera exigua* and *S. frugiperda*. The average outcome case scenario leads to an overall probability of zero (therefore no probability distribution could be built) since it assumed successful temporal overlap (*p* = 1) but no spatial overlap (*p* = 0).

**Figure 4:**
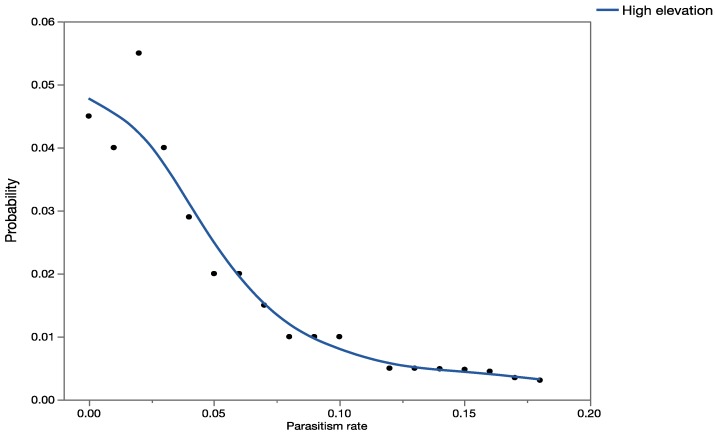
Forecast of all possible parasitism rates (as proportion on the *x*-axis) for *C. marginiventris* at high-elevation sites using field-collected data from retrospective studies in Hawaii.

In Hawaii, *C. marginiventris* occurs in forest and scrubland/shrubland areas, and therefore overlaps with some populations of *U. stellata*, consistent with the assumptions in the hypothetical worst-case outcome scenario. Figure 4 shows the forecasted probability of parasitism rates of *U. stellata* in Hawaii at high-elevation sites. Parasitism of *U. stellata* by *C. marginiventris* was not recorded at low- and medium-elevation sites in Hawaii, even though the average outcome case scenario predicted that this parasitoid could also occur at low and medium elevations.

The Kruskal–Wallis ANOVA on ranks was used to test for significant differences in forecasts from the statistical reports of the Monte Carlo (MC) simulations analyses with data gathered from the literature at the three elevations. The Mann–Whitney Rank Sum Test was used to detect significant differences between results of the MC analysis with historical data and data from current studies.

There were significant differences in parasitism rates by *C. marginiventris* reported in the literature at the three elevations studied (H = 2255.2; *p* < 0.001). When results of the MC analysis with parasitism rates recorded at high elevations in Hawaii were compared statistically with results of the MC simulation analysis of parasitism from older literature for low, medium, and high elevations, observed field parasitism of *U. stellata* at high elevations in Hawaii was significantly higher than predicted parasitism of *S. exempta* at low-, medium-, and high-elevation sites (*p* < 0.001).

Based on the results of the simulation with the historical published data (which represents the predicted worst-case scenario), parasitism rates of *U. stellata* by *C. marginiventris* in Hawaii were expected to be higher at medium- and low-elevation sites and minimal at high-elevation sites. Yet, recent quantitative data from field studies in Hawaii show that all parasitism of *U. stellata* by *C. maginiventris* occurred at high elevations and none at low and medium elevations. The target hosts *S. exempta* and *S. frugiperda* are distributed from low- to high-elevation sites in the area where data on parasitism rates by *C. marginiventris* were gathered from Mexico.

Based on historical published records, it would have been likely to predict that *U. stellata* would be within the physiological host range of *C. marginiventris*. It would also have been possible to predict that this species should be able to persist across a long elevation gradient. However, the published records did not provide any indication that this species will be able to occupy areas other than agricultural land and grassland, but of course this is an artifact of the data being limited to agricultural areas. Should data have been available for higher elevation areas, the predicted scenario would possibly have been different.

#### 2.1.2. *Meteorus laphygmae*

Table 7 shows a list of hosts of *M. laphygmae* known prior to its introduction to Hawaii. Most literature records associate *M. laphygmae* with Noctuidae pests in agricultural systems. Based on the records available at the time of introduction, there was no indication that this species would attack species in the family Crambidae. Subsequent records of non-target parasitism in Hawaii have shown that this species can attack hosts in the families Crambidae, Oecophoridae, Tortricidae, and Heliconiidae, and non-target hosts in the families Noctuidae and Geometridae (Table 2).

**Figure 5:**
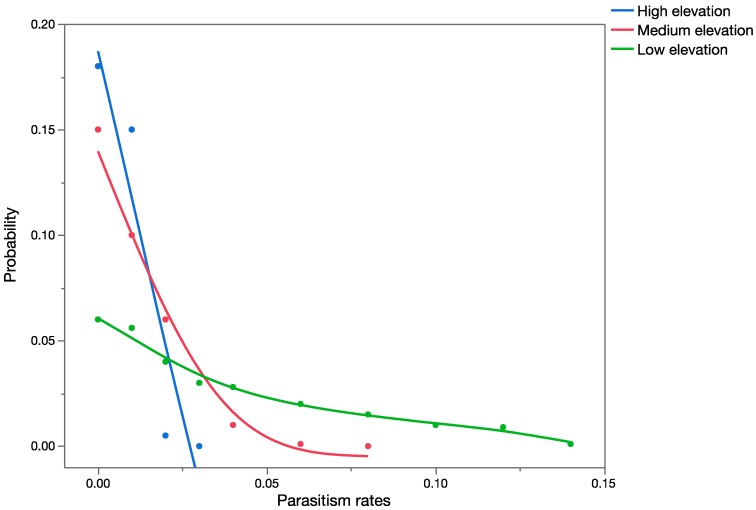
Overlay chart showing a forecast of all possible parasitism rates (as a proportion on the *x*-axis) for *M. laphygmae* at low-, medium-, and high-elevation sites using published data.

**Figure 6:**
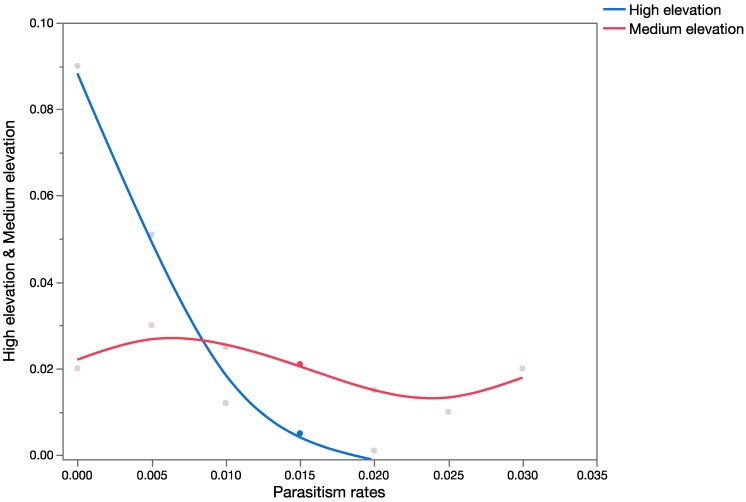
Overlay chart showing a forecast of all possible parasitism levels (as proportion on the *x*-axis) for *M. laphygmae* at medium- and high-elevation sites using field-collected data from Hawaii.

As with *C. marginiventris*, *M. laphygmae* was predicted to occur only in grassland and agricultural areas. The worst-case scenario assumed that *M. laphygmae* could occupy habitats of *U. stellata* and parasitize hosts in the family Crambidae at the same parasitism rates as their target hosts *S. exigua* and *S. frugiperda* in Mexico. Retrospective studies in Hawaii [33,34] have shown that *M. laphygmae* can occupy the same habitats as *U. stellata* and successfully parasitize this non-target species. Figure 5 shows results of the PRA analysis using data from the historical literature for low-, medium-, and high-elevation sites. Figure 6 shows the probability distributions for the observed parasitism rates on *U. stellata* in Hawaii at medium- and high-elevation sites. *Meteorus laphygmae* was not found parasitizing the larvae of *U. stellata* at low-elevation sites.

There were significant differences in parasitism rates by *M. laphygmae* reported in the historical data at the three elevations studied (H =1725.8; *p* < 0.001). When results of the simulation analysis with current data from Hawaii at medium- and high-elevation sites were compared statistically, parasitism rates at high elevations were significantly higher than at medium-elevation sites (*t* = 4,978,341; *p* < 0.001). There was no significant difference between the results of the simulation analysis and the historical published parasitism data at low elevations, and the results of the simulation analysis with parasitism on *U. stellata* in Hawaii at high-elevation sites using current data (*t* = 3,808,532; *p* = 0.444). No significant differences were found between the results of the simulation with data from the literature at medium elevation and results with current data from Hawaii at medium elevations (*t* = 3,850,017; *p* = 0.886). *Meteorus laphygmae* can inflict as much parasitism on *U. stellata* as on its target hosts, but this parasitism occurs at altitudes where the target pest hosts are not present.

Based on published historical records, it would have been unlikely to predict that *U. stellata* would be in the physiological host range of *M. laphygmae,* since there was no indication that this species would attack hosts in the family Crambidae. It would have been possible to predict that this species would occur across a wide range of elevations. The expectation that *M. laphygmae* would inflict similar parasitism levels on a non-target species as on its preferred hosts in Hawaii did not hold valid: it would have been predicted that higher parasitism rates would be observed at low-elevation sites rather than medium and high elevations. No parasitism by *M. laphygmae* was detected at low elevations, but only at medium and mostly high elevations. As in the case of *C. marginiventris*, the target hosts of *M. laphygmae* in Hawaii such as *Herpetogramma licarsisalis*, *S. mauritia* (pests of turf grasses), *S. exempta* (a pest of sugar cane and grasses), and *S. exigua* (a common pest of cruciferous crops) predominate at low and medium elevations, which may suggest that the degree of non-target parasitism might be mediated by the availability of preferred hosts. Duan et al. [68] reported similar results from a different assemblage of parasitoids and hosts in Hawaii—the lantana gall fly (a purposely introduced biological control agent of a *Lantana camara*, and subsequently a non-target host species) was more heavily parasitized by the fruit fly parasitoid *Dichasmimorpha tryoni* in upland forest habitats rather than in low-land pastures and mid-elevation sugarcane fields. 

#### 2.1.3. *Trathala flavoorbitalis*

No host records could be obtained for *T. flavoorbitalis* from the native provenance prior to the year that it was first observed in Hawaii. There are reports on its hosts in its place of origin from later dates. Table 3 shows the recorded hosts in Hawaii.

**Figure 7:**
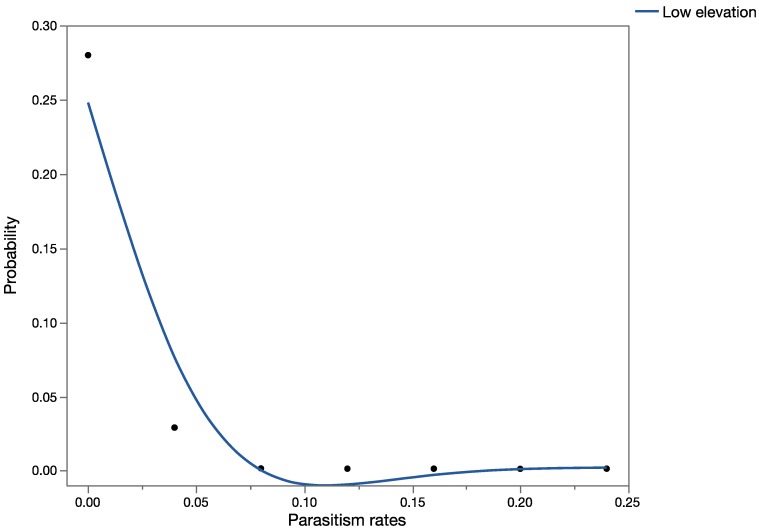
Forecast of all possible parasitism rates (as a proportion on the *x*-axis) for *T. flavoorbitalis* at low-elevation sites using published data.

**Figure 8:**
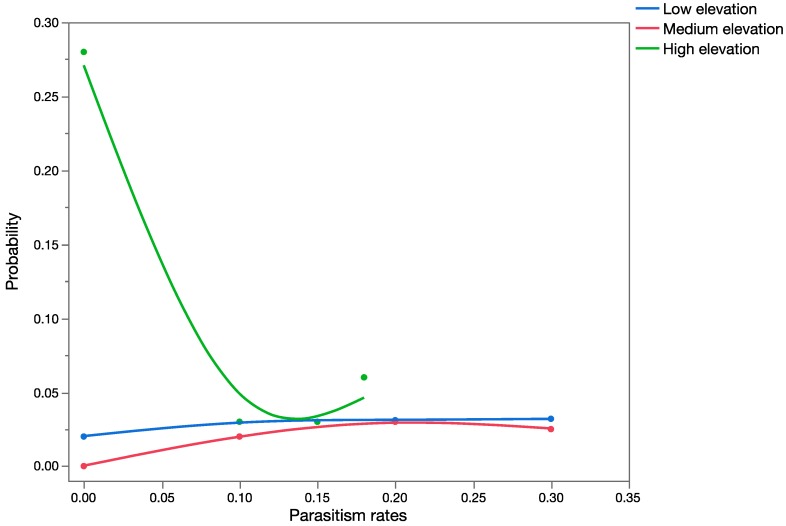
Overlay chart, showing forecast of all possible parasitism rates (as a proportion on the *x*-axis) for *T. flavoorbitalis* at low-, medium-, and high-elevation sites using data from retrospective studies in Hawaii.

This species was found parasitizing *U. stellata* in Hawaii at low-, medium-, and high-elevation sites, in contrast with the historical literature, which only reports parasitism in low-elevation agricultural areas in the native range. The worst-case scenario assumed that this species would occupy *U. stellata* habitats and attack this non-target species at the same rates as its known target host, *Antigastra catalaunalis* (Lepidoptera: Crambidae), in India. Figure 7 shows the predictions of the probabilistic analysis using published historical data, at low-elevation sites. Figure 8 shows the results of the MC analyses with field parasitism rates of *U. stellata* at low-, medium-, and high-elevation sites in Hawaii. When results of the simulation analysis using published historical data at low elevations were compared statistically with the results of the simulation analysis using field data in Hawaii at low-elevation sites, field parasitism in Hawaii was significantly higher (*t* = 5,418,198.5; *p* < 0.001). When the results of the simulation analysis with retrospective data from Hawaii at three elevations were compared statistically using the Kruskal–Wallis ANOVA on ranks, significant differences were found (H = 2517.7; *p* < 0.001): parasitism rates at low and medium elevations were significantly higher than parasitism rates at high-elevation sites (*p* < 0.05).

Based on these comparisons, using published host records would have been inadequate to predict that *T. flavoorbitalis* would occur at medium and high elevations in Hawaii. Known target hosts from its area of distribution (*Spoladea recurvalis* and *Herpetogramma licarsisalis*) are also present at low elevations in Hawaii, yet significantly higher parasitism rates on *U. stellata* by *T. flavoorbitalis* occurred at low and medium elevations than at high elevations, which contrasts with the pattern seen with *C. marginiventris* and *M. laphygmae*. Perhaps this species performs better at lower elevations and/or the known hosts in the area of introduction are not preferred hosts.

### 2.2. Question 3: How Useful Are Estimates of Marginal Attack Rates versus Apparent Mortality (and Measures of Uncertainty in Both) in Conducting Probabilistic Risk Assessment?

**Figure 9:**
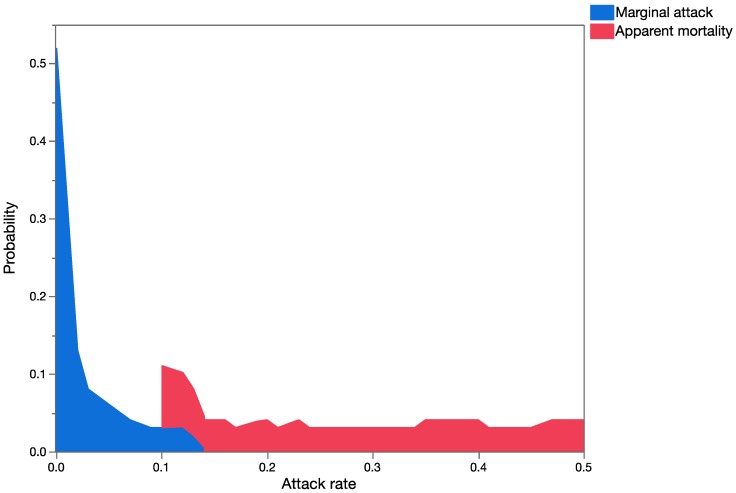
Overlay chart showing the probability distributions of marginal attack rate and apparent mortality (expressed as proportions on *x*-axis) for *T. flavoorbitalis*.

**Figure 10:**
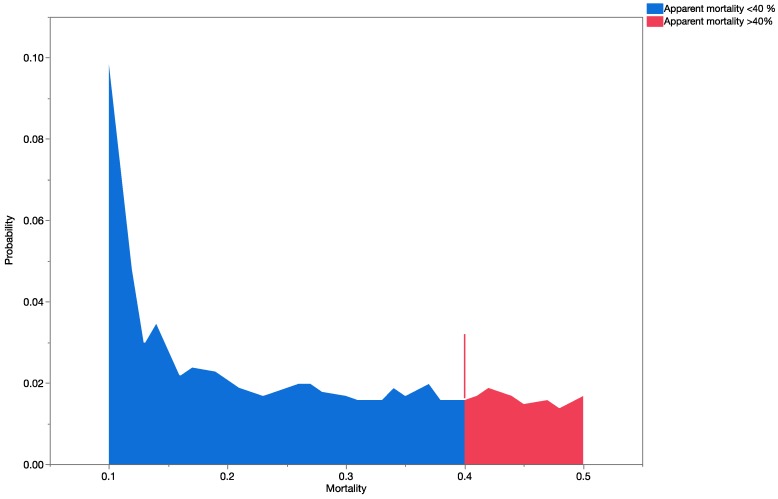
Distribution of all possible values of apparent mortality rate for *T. flavoorbitalis*, specifically indicating a 28.21% probability that expected parasitism values will rise above 0.40.

No probability distributions could be built for *C. marginiventris* and *M. laphygmae* marginal attack rates since 17 of the 18 values based on actual field data were zero (Table 7). Figure 9 and Figure 10 show a graphical summary of the output of the Monte Carlo simulations for *T. flavoorbitalis* for marginal attach rates and apparent mortality, respectively. The range of apparent mortality rates varied from zero to 59%, whereas the range of possible outcomes using marginal attack rate ranged from zero to 15%. Results of the Mann–Whitney Rank Sum Test showed that there were significant statistical differences (*t* = 1,960,261; *p* < 0.001) in the outputs of the simulation analysis using the apparent mortality and marginal mortality datasets. Simulations using apparent mortality significantly increased the estimates of risk. Results of the MC analyses with apparent mortality data were still significantly higher than the results with marginal mortality data when a second uncertainty factor (representing ‘spatial overlap’) was added to the analysis (*p* < 0.0001; using sets 3, 4, and 10 in Table 6). Figure 10 shows the range of all possible values for *T. flavoorbitalis* apparent mortality, specifically indicating a 28.21% probability that values of apparent mortality will fall above 40%, the approximate threshold value for a natural enemy to exert control on a population [69].

The use of marginal attack rates derived from life table studies should be encouraged whenever possible as they provide a more realistic estimate of the non-target risk, because they indicate the level of generational mortality, whereas apparent mortality does not have population impact context and can potentially overestimate or underestimate the actual level of mortality in situations where susceptible stages are over- or under-sampled [70,71,72].

### 2.3. Question 4: Are There Any Key Ecological Variables That Would Be Worth Considering in the Hypothetical Case that Any of the Species Might Be Considered for Introduction in Another Place, or Are Worth Considering in Any Risk Assessment?

Environmental context clearly influenced the level of parasitism of *U. stellata* in this study system. Results of this work, and previous papers, have shown that the parasitism incurred by *U. stellata* is different at low, medium, and high elevations. In the case of *T. flavorbitalis*, parasitism rates on *U. stellata* at low- and medium-elevation sites were significantly higher than at high-elevation sites. Parasitism of *U. stellata* by *C. marginiventris* and *M. laphygmae* was significantly higher at high elevations than at medium or low elevations. If non-target species of interest occur in multiple habitats or across a wide range of environmental conditions, such as elevation and level of disturbance by alien plants [36], knowledge of parasitism under those conditions should be incorporated in the PRA. 

Even though it has been shown that non-target parasitism varies between habitats [32,72,73,74], most risk assessment studies place emphasis on assessing the host range or level of non-target parasitism, but not on assessing how habitat and/or environmental/ecological gradients may mediate those levels of non-target parasitism. By incorporating spatial and ecological context in risk assessment, it may be possible to identify habitats that are likely to be at higher risk of adverse effects, and use that information for regulatory decisions. For example, if parasitism rates on a non-target species are expected to occur, or be above an acceptable level in habitats in conservation areas, the introduction of the BC agent might not be justified. Conversely, if non-target parasitism is expected in areas that do not have conservation priority, like highly degraded habitat invaded by alien plants, the decision might depend on the level of confidence that parasitism levels will be below a certain acceptable level (which will not cause an impact at the population level). Results from this type of risk assessment can also provide a foundation for the establishment of possible management programs. For example, when considering augmentative biological control agents, depending on the habitat and the expected level of parasitism there, it could be possible to restrict releases to areas that have low predicted levels of risk. Furthermore, by incorporating data centered on seasonal effects, it could be possible to plan augmentative releases at times in the year when they pose lower levels of risk to non-target species.

Another component that likely influenced the degree of non-target host use in this study system was the availability of preferred hosts. In the case of oligophagous or polyphagous species, the presence of preferred and/or alternative hosts can play an important role in the level of non-target attack [75,76,77]. *Cotesia marginiventris* and *M. laphygmae* can occur in a wide range of ecological conditions [58,78]; nevertheless, their association with *U. stellata* is mainly in less disturbed, high-altitude sites in Hawaii, which are dominated by native species. The fact that they are associated with *U. stellata* at high-altitude sites does not necessarily mean that they prefer those habitats, but perhaps that at those sites they exploit endemic non-target species due to the absence of their preferred hosts. It is therefore important to not only describe the environment that the biocontrol candidate and non-target species inhabit, but also the environment that the target hosts will inhabit in the area of introduction, since BC agents can disperse beyond the habitats of the target hosts [31].

## 3. General Discussion

The major advantage of the PRA framework is that it can be designed to consider effects to non-target species at a spatial scale and takes uncertainty in datasets into consideration, and thus evaluates the risks posed by new introductions in a more ecologically meaningful way than procedures that rely on single-point (typically mean) estimates of parasitism, and that do not take spatial variability into account. Single point estimates ignore variation in a dataset, and provide a false sense of precision that leads to what is called “analysis paralysis” [60]. More realistic and meaningful procedures are not always the simplest to apply, though. PRA will require more data than other proposed and current approaches for assessing non-target risks [24,25], and these data may not always be available for researchers and decision makers. It is not necessary to use probability distributions for all input variables in a PRA, but the effectiveness and robustness of risk estimates is increased if one does use them. A precise and realistic model will necessarily be limited to a few applications, whereas a very general approach applicable to many situations will almost certainly be limited in precision.

Not all non-target species are affected equally, and the same non-target species may not be affected equally under different environmental conditions [32,73]. Comprehensive risk assessment requires a wide-ranging understanding of the ecology of the biological control agent, as well as the ecology of the target and non-target species [78,79]. Observations on the ecology of the biocontrol agent in other areas of introduction and the area of origin, and how parasitism rates vary in those areas under different environmental/ecological conditions, can provide useful baseline information for predictions made for new locations.

Biological control practitioners and regulators prefer the release of agents with narrow host ranges [77]. Nevertheless, when faced with situations where agents have great potential to control the target pest but may attack some native and beneficial species, careful characterization of the risk should be done to estimate the relative impact of the proposed introduction on populations of the non-target hosts and weighted against the potential benefits [80,81]. In those situations PRA could be a tool for better decision-making. Some retrospective studies on polyphagous biocontrol species have shown that they cause minimal impact on the non-target species, underscoring the importance of carefully estimating population-level impacts [82,83]. It would be valuable to weigh the environmental benefits attributable to those releases that pose some level of risk, to determine if the non-target parasitism on endemic species, even if minimal, was justified. Additive negative impacts such as habitat destruction and mortality from multiple sources may result in even minimal non-target mortality becoming important; therefore, it is also important to assess the possible impacts of biocontrol introductions in relation to other current sources of mortality [73].

## 4. Conclusions

Forecasts from risk assessment analysis may be used to make decisions to either accept or reject a proposed biocontrol introduction. As far as possible, quantitative methods that incorporate measures of uncertainty should be chosen. Consideration of the uncertainty in probabilistic analysis can be used to set standards based on the probability that the threshold for unacceptable effects will be exceeded [84], and therefore not only provides information for the risk characterization phase but can play an important role in a risk-benefit analysis phase.

Not all proposed introductions would need PRA; clearly safe BC agents—such as those that demonstrate no non-target attack in screening trials—will not require such an assessment. The environmental risk assessment proposed by van Lenteren et al. [24,25] can be used as a first step to encourage the screening of safe biological control agents to all potential non-target species using pre-determined qualitative and quantitative rankings. When promising BC agents are found to attack non-target species, PRA has the potential to provide an effective means to characterize the risk to selected non-target species of concern, which can be used to estimate the degree of confidence that a certain level of non-target effects will not be exceeded, and could further be implemented in risk-benefit analysis. Non-target effects from biological control introductions are not desirable, but if minimal (not causing a significant impact at the population level) effects are predicted with high levels of confidence, the decision to accept or reject a BC agent can be knowledge-driven rather than fear-based [77].

## Figures and Tables

**Table 1:** List of known hosts of *C. marginiventris* in Hawaii (adventive = accidental introduction).

Species	Family	Origin	Reference
*Agrotis hephaestaea* (Meyrick)	Noctuidae	Endemic	[49]
*Ethmia nigroapicella* (Saalmuller)	Oecophoridae	Adventive	[48]
*Eudonia* sp.	Crambidae	Endemic	[49]
*Eupithecia monticolens* Butler	Geometridae	Endemic	[49]
*Fletcherana leucoxyla* Meyrick	Geometridae	Endemic	[49]
*Haliophyle euclidias* Meyrick	Noctuidae	Endemic	[49]
*Scotorythra arboricolans* Butler	Geometridae	Endemic	[49]
*Scotorytorythra caryopis* Meyrick	Geometridae	Endemic	[48]
*Scotorythra hecataea* Meyrick	Geometridae	Endemic	[49]
*Scotorythra pauludicola* (Butler)	Geometridae	Endemic	[48]
*Scotorythra rara* Butler	Geometridae	Endemic	[49]
*Scotorythra trapezias* Meyrick	Geometridae	Endemic	[48]
*Scotorythra* spp.	Geometridae	Endemic	[49]
*Spodalea recurvalis* (Fabricius)	Crambidae	Adventive	[48]
*Pseudaletia unipuncta* (Haworth)	Noctuidae	Adventive	[48]
*Spodoptera exempta* (Walker)	Noctuidae	Adventive *	[48]
*Spodoptera mauritia* (Boisduval)	Noctuidae	Adventive	[48]
*Spheretista pleonectes Walsingham*	Tortricidae	Endemic	[49]
*Udea stellata* (Butler)	Crambidae	Endemic	[33]

* Target host.

**Table 2:** List of known hosts of *M. laphygmae* in Hawaii (adventive = accidental introduction, Introduced = purposely introduced).

Species	Family	Origin	Reference
*Agraulis vanillae* (Linnaeus)	Nymphalidae	Adventive	[49]
*Agrotis hephaestaea* (Meyrick)	Noctuidae	Endemic	[48]
*Agrotis ipsilon* (Hufnagel)	Noctuidae	Adventive *	[49]
*Amorbia emigratella* Busck	Tortricidae	Adventive	[49]
*Amyna natalis* (Walker)	Noctuidae	Adventive	[49]
*Autegumia ebulealis* Guenee	Crambidae	Introduced	[49]
*Elaphria nucicolora* (Guenee)	Noctuidae	Adventive	[49]
*Eudonia* sp.	Crambidae	Endemic	[49]
*Eupithecia monticolens* Butler	Geometridae	Endemic	[49]
*Fletcherana leucoxyla* Meyrick	Geometridae	Endemic	[49]
*Haliophyle euclidias* Meyrick	Noctuidae	Endemic	[49]
*Helicoverpa zea* (Boddie)	Noctuidae	Adventive	[48]
*Herpetogramma licarsisalis* (Walker)	Crambidae	Adventive	[48]
*Melipotis indomita* (Walker)	Noctuidae	Adventive	[48]
*Omiodes accepta* (Butler)	Crambidae	Endemic	[48]
*Rynchopalpus brunellus* Hampson	Noctuidae	Introduced	[48]
*Scotorythra* spp.	Geometridae	Endemic	[49]
*Scotorythra apicalis* Swezey	Geometridae	Endemic	[49]
*Scotorythra hecataea* Meyrick	Geometridae	Endemic	[49]
*Scotorythra ortharcha* Meyrick	Geometridae	Endemic	[49]
*Scotorythra rara* Butler	Geometridae	Endemic	[49]
*Scotorythra paludicola* (Butler)	Geometridae	Endemic	[48]
*Scotorythra trapezias* (Meyrick)	Geometridae	Endemic	[48]
*Spodoptera exempta* (Walker)	Noctuidae	Adventive *	[48]
*Spodoptera exigua* (Hubner)	Noctuidae	Adventive *	[48]
*Spodoptera mauritia* (Boisduval)	Noctuidae	Adventive *	[48]
*Thyracopa* spp.	Oecophoridae	Endemic	[49]
*Udea stellata* (Butler)	Crambidae	Endemic	[48]
*Udea pyranthes* (Meyrick)	Crambidae	Endemic	[49]

* Target host.

**Table 3:** List of known hosts of *T. flavoorbitalis* in Hawaii (adventive = accidental introduction, Introduced = purposely introduced; ? = uncertain) [55].

Species	Family	Origin
*Agonoxena argaula Meyrick*	Agonoxenidae	Adventive
*Asymphorodes dimorpha* (Busck)	Asymphorodes	Aventive
*Bradleyella metallurgica* (Walsingham)	Tortricidae	Endemic
*Bracta straminea* (Butler)	Tortricidae	Adventive?
*Bracta venosana* (Zeller)	Tortricidae	Introduced
*Carposina gramicolor* (Walsingham)	Carposinidae	Endemic
*Chedra microstigma* (Walsingham)	Batrachedridae	Adventive
*Chilo supressalis* (Walker)	Crambidae	Adventive
*Crocidosema blackburni* (Butler)	Tortricidae	Endemic?
*Crocidosema marcidella* (Walsingham)	Tortricidae	Endemic?
*Crocidosema lantana* Busck	Tortricidae	Introduced
*Cryptophlebia illepida* (Butler)	Tortricidae	Adventive
*Erechthias minuscula* (Walsingham)	Tineidae	Adventive
*Herpetogramma licarsisalis* (Walker)	Crambidae	Adventive
*Omiodes accepta* (Butler)	Crambidae	Endemic
*Omiodes blackburni* (Butler)	Crambidae	Endemic
*Omiodes localis* (Butler)	Crambidae	Endemic
*Omiodes meyricki* Swezey	Crambidae	Endemic
*Omiodes monogramma* Meyryck	Crambidae	Endemic
*Omiodes muniscola* Swezey	Crambidae	Endemic
*Pyroderces rileyi* (Walsingham)	Cosmopterigidae	Adventive
*Spheterista infaustana* (Walsingham)	Tortricidae	Endemic
*Spheterista reynoldsiana* (Swezey)	Tortricidae	Endemic
*Spodalea recurvalis* (Fabricius)	Crambidae	Adventive
*Thyrocopa* spp.	Oecophoridae	Endemic
*Udea chalcophanes* (Meyrick)	Crambidae	Endemic
*Udea micacea* (Butler)	Crambidae	Endemic
*Udea platyleuca* (Meyrick)	Crambidae	Endemic
*Udea stellata* (Butler)	Crambidae	Endemic
*Udea violae* (Swezey)	Crambidae	Endemic

**Table 4:** Apparent mortality data used in simulations for Question 3. (Data from [35]).

Datum	*Trathala*	*Meteorus*	*Cotesia*
1	0.106	0.070	0.088
2	0.141	0.001	0.001
3	0.303	0.059	0.176
4	0.122	0.001	0.001
5	0.489	0.001	0.083
6	0.510	0.001	0.001
7	0.243	0.001	0.001
8	0.585	0.001	0.001
9	0.338	0.001	0.001
10	0.222	0.001	0.001
11	0.411	0.001	0.001
12	0.508	0.001	0.001
13	0.295	0.030	0.091
14	0.373	0.001	0.001
15	0.174	0.001	0.071
16	0.538	0.001	0.001
17	0.333	0.001	0.001
18	0.146	0.001	0.001
19	0.430	0.001	0.001
20	0.600	0.001	0.026
21	0.123	0.001	0.001
22	0.103	0.014	0.014
23	0.152	0.001	0.001
24	0.144	0.001	0.266
25	0.193	0.001	0.302

**Table 5:** Marginal attack rate data used for simulations in Question 3. (Data from [34]).

Datum	*Trathala*	*Meteorus*	*Cotesia*
1	0.006	0.006	0.000
2	0.040	0.000	0.000
3	0.015	0.000	0.000
4	0.001	0.000	0.000
5	0.001	0.000	0.017
6	0.053	0.000	0.000
7	0.001	0.000	0.000
8	0.001	0.000	0.000
9	0.001	0.000	0.000
10	0.128	0.000	0.000
11	0.037	0.000	0.000
12	0.035	0.000	0.000
13	0.080	0.000	0.000
14	0.009	0.000	0.000
15	0.001	0.000	0.000
16	0.017	0.000	0.000
17	0.141	0.000	0.000
18	0.031	0.000	0.000

**Table 6:** List of hosts of *C. marginiventris* in its native range known prior to its introduction to Hawaii in 1942.

Species	Family	Reference
*Pseudaletia latiuscula* (Herrich-Schaffer)	Noctuidae	[61]
*Spoladea recurvalis* (Fabricious)	Crambidae	[62]
*Spodoptera exigua* (Hübner)	Noctuidae	[63]
*Spodoptera frugiperda* (Smith)	Noctuidae	[64]
*Spodoptera praefica* (Grote)	Noctuidae	[65]
*Udea rubigalis* (Guenee)	Crambidae	[66]

**Table 7:** List of known hosts of *M. laphygmae* in its native range prior to its introduction to Hawaii in 1942.

Species	Family	Reference
*Agrotis subterranea* (Fabricius)	Noctuidae	[43]
*Helicoverpa zea* (Boddie)	Noctuidae	[43]
*Monodes* spp.	Noctuidae	[43]
*Pseudaletia unipuncta* (Haworth)	Noctuidae	[67]
*Spodoptera* spp.	Noctuidae	[43]
*Spodoptera praefica* (Grote)	Noctuidae	[66]
*Spodoptera frugiperda*(J. E. Smith)	Noctuidae	[43]
*Spodoptera exigua*(Hübner)	Noctuidae	[43]
*Colias eurytheme* (Boisduval)	Pieridae	[43]

## Supplementary Materials

The supplementary materials are available online at http://www.mdpi.com/2075-4450/8/3/67/s1.
