# Supplementary material for: Assessing Probabilistic Risk Assessment Approaches for Insect Biological Control Introductions
Source: Insects. 2017 Jul 7;8(3):67. doi: 10.3390/insects8030067 (PMC5620687; doi:10.3390/insects8030067)
Supplement: Supplementary file 1 [file insects-08-00067-s001.pdf]

Insects **2017**, *8*, 67; doi:10.3390/insects8030067 www.mdpi.com/journal/insects

Supplementary Materials: Assessing Probabilistic Risk Assessment Approaches for Insect Biological Control Introductions—Overview and Critique of Proposed Methodologies for Risk Assessment for Insect Biological Control

Leyla V. Kaufman and Mark G. Wright

1. Environmental Risk Assessment

Van Lenteren et al. [1] proposed an environmental risk assessment protocol for biological control agents used for inundative release programs. This first general framework was based on a semi-quantitative system that integrates information on potential of establishment, dispersal, host range, direct effects, and indirect effects. Van Lenteren et al. [2,3] later proposed a refined framework for both augmentative and classical biological control programs that distinguish between native and exotic agents in a stepwise system. Van Lenteren et al.'s approach includes seven steps. In the first two steps the risk assessor distinguishes between native and exotic natural enemies as well as the type of biological control program intended. The other steps refer to each the risk components in the following order: establishment, host range, dispersal, and direct and indirect effects.

The van Lenteren approach uses predetermined qualitative and quantitative scales to describe the likelihood and magnitude for each of the five risk components identified to calculate risk indexes. In the first proposed approach [1] a numerical value (1 to 5) was added to the each descriptor of likelihood and magnitude, thereafter the calculation of the overall risk was done by first multiplying the respective numerical value for likelihood and magnitude for each of the five risk components and subsequently summing those scores. Once the overall risk index is calculated, it is ranked based on a threshold scale that classifies the risk into low, intermediate, or high-risk categories. The authors identified some shortcomings of this approach: information about likelihood and magnitude for each of the risk components had to be available before the evaluation, even for obviously risky agents, causing unnecessary research. Also, summing scores of risk components that are not completely independent from each other (most components are conditional events) was considered inadequate, therefore in the refined approach [2,3] weighting factors are provided for the calculation of the environmental risk index (ERI) for the following risk components: establishment, dispersal and direct and indirect effects. If the ERI falls above a given threshold for each of the risk components, the RA is recommended to be discontinued at that step, or it may continue upon request.

The main purpose of the stepwise approach is early identification of prospective species that pose a high risk of negative direct and indirect effects; these species are eliminated early in the process, avoiding unnecessary further research. The van Lenteren stepwise framework guides the risk assessor throughout the risk analysis process and clearly distinguishes between augmentative and classical biological control agents, as well as native and exotic agents. The strength of this approach is that it is possible to use qualitative and quantitative information. In many situations, quantitative data are difficult to obtain, making a framework that incorporates qualitative information more flexible. When assessing the risk of augmentative species already in use, the quick scan method (which differs from the full stepwise assessment in that information does not need to be generated but is already available) promotes the continuation of successful programs and the discontinuation of programs that are too risky for certain ecoregions. A full risk assessment is only necessary for new species.

One of the main risk components of this approach is establishment in a non-target habitat, which is only taken into consideration for augmentative biological control (ABC) agents and not for classical biological control (CBC) agents. Even though the main difference of both BC programs is

establishment, when this happens in a non-target habitat then it is not desirable for either augmentative or classical BC.

One weakness of this approach is that the risk components identified by the authors are indeed important, but they are not used in an integrated way. In this system, the steps are not in a logical sequence of events, which is evident when assessing the risk of an inundative biological control agent. The host range issue is considered before dispersal to suitable habitats. In a situation where there is no chance of establishment, dispersal to potential suitable habitats (where potential non-targets may be present) will play a significant role in determining the ecological host range. Following this framework, agents that do not have restricted diets may be eliminated early in the process (or it may continue upon request of the applicant) based on their fundamental host range without consideration of filters such the ability to disperse to habitats where non-targets may be exposed to the agent. Therefore, it may be possible to fall into the bias of eliminating agents that do not pose a real risk if exposure is not clearly evaluated. This approach is therefore centered on the premise that eliminating risky species early in the process based primarily on their fundamental host range can save resources. Nevertheless, even when host range indicates that valued species can be used as non-target hosts, the agent may still cause no or low risk of impact at the population level [4–7]. Further research on promising agents can therefore be justified if the benefits outweigh the risks. Also, this approach is used to infer the risk posed by an introduction to all non-target species, which loses resolution on the level at which each non-target species is affected.

Finally, this approach lacks incorporation of uncertainty. Suter [8] highlighted three drawbacks of the use of single-point deterministic approaches, which consider only an average estimate of a variable, rather than incorporating a measure of variability: (1) it is inconsistent (because it can hide inherent variability); (2) conservative assumptions tend to hide uncertainties and error from the decision maker by burying it in estimates of exposure and effects; and (3) conservatism assumes that there are no societal environmental costs of regulation erroneously based on false positives.

1.1. Probabilistic Risk Assessment (PRA) for Biological Control Introductions

This approach was proposed by Wright et al. [9] and is based on Bayesian reasoning to predict risk posed by prospective biological control agents to non-target species. Probabilistic risk assessment (PRA) uses probability distributions to characterize variability or uncertainty in risk estimates. Note that the ability to perform a PRA often is limited by the availability of probability distributions that adequately describe one or more of the input parameters. The quantitative analysis of uncertainty or variability can provide a more comprehensive characterization of risk than conservative approaches which rely on single point estimates. Results of a PRA provide a range of all possible outcomes (e.g. levels of parasitism) and their likelihood of occurrence, therefore allowing one to ask “what if” questions.

Event trees (also known as precision trees or fault-trees) are appropriate for displaying the order of events that result in the ‘risk event’ being realized, and dependency between them (conditional probability) [10]. By modeling the possible events that can occur after the release of a biological control agent, the risk assessor may identify potential routes of risk that need to be quantified. Event trees consist of a network of nodes and connecting branches, and outcomes of occurrences. Nodes indicate decision points and chance events. Branches correspond to the impact of each decision alternative or event outcome emerging from a node.

The use of the PRA approach in biological control requires quantification of key biological and ecological factors playing a role in non-target impacts, estimation of probability distribution to express variation for relevant factors measured, and articulation/construction of the precision tree in the appropriate sequence of events. The overall probability of impact from a series of events is estimated by multiplying the P values along each branch.

Wright et al. [9] used the augmentative egg parasitoid *Trichogramma ostrinae* Pang and Cheng (Hymenoptera: Trichogrammatidae) as a case study. This parasitoid is unable to survive winters in the northeastern United States. For the construction of the precision trees they considered aspects such as physiological host range of the parasitoid, dispersal capacity within and out of cornfields (the

target release habitat), searching and host-location behavior in corn and crops of different architectures, and searching efficiency in indigenous deciduous forests. The authors underscored the importance of including key ecological traits into the RA to adequately estimate the probability of an adverse effect, rather than relying only on laboratory screening which only provides information on potential physiological host range, not potential ecological/realized host range. In their case study, they presented a simplified application of probabilistic analysis, presenting only two possible outcomes (the average and the worst case scenario, based on probability distributions) instead of a range of all possible outcomes.

Uncertainty is a fundamental characteristic of RA. Uncertainty may arise from unknown data or incomplete knowledge as well as from natural variability in a dataset. In decision tree or Bayesian statistical analysis there are different sequences of events that could contribute to the overall risk, therefore there is a need to assess how the estimate of risk is affected by uncertain components. An accurate prediction of the risk is therefore a daunting task. Uncertainty analysis provides a range of risk values that could be taken into consideration to evaluate risk under different scenarios.

Among the most important strengths of this approach is the incorporation of uncertainty in the analysis. Modeling different scenarios allows the risk assessor to answer "what if" questions. This aspect could be valuable for the risk benefit analysis phase. An additional strength of this approach is the incorporation of ecological data into the analysis that optimizes the estimate of risk posed by the introduction. Contrary to the environmental risk assessment approach [2,3], this approach cannot be used to infer the risk to all non-target species but only to selected species selected for scrutiny. This approach has the potential to produce high resolution predictions regarding the extent to which effects may occur for a specific species of concern.

The primary disadvantages of PRA are that it generally requires more time, resources, and expertise on the part of the assessor and reviewer than a point estimate approach, and therefore should be conducted only in cases where the benefits are thought to outweigh the risks. Probability distributions of the different events are often difficult to obtain which can therefore limit the use of this approach. The proposed framework lacks incorporation of spatial and temporal components to characterize the risk. A clear incorporation of spatial and temporal components can provide insights about areas that are at low or high risk of non-target effects, information that can be used in the risk management phase. The Wright et al. [9] approach was proposed using an augmentative biological control agent already in use as a case study, therefore the generation of required ecological data such as dispersal outside the release area and host searching in different habitats was readily collected. The use of this approach as a predictive tool for new species will be limited to information that is available in the current literature from other places and in information generated from rigorous field studies in the country of origin. The proposed approach had no generally defined structure as far as phases in the problem formulation, analysis phase and risk characterization, conditions that make this approach difficult for widespread adoption.

2. Refined Probabilistic Risk Assessment

PRA should be conducted in situations where the proposed candidate has demonstrated great potential for controlling the target pest and when potential non-target hosts have been identified to be species of concern based on results of host specificity testing in the laboratory. PRA is then directed to estimate the probability that effects on selected non-target species will materialize and the magnitude of those effects. Phases to follow in PRA are problem formulation, analysis phase, and risk characterization.

2.1. Problem Formulation

This is a fundamental component of any risk assessment [11,12]. The problem formulation should include the following information:

- Description of the biological control agent (stressor). Description of the basic biology, life cycle, known host range and other relevant information. The determination of the fundamental host

range can be determined from literature records and/or during field surveys in the agent's country of origin.
- Non-target hosts (assessment endpoints) considered as of concern are selected for PRA. These are those entities that represent economic, ecological or cultural value for society and that are to be protected, and are selected based on their susceptibility to the biological control agent (stressor) [11,12]. Species are selected based on results from host specificity studies and high likelihood of being used as non-target hosts under field conditions.
- Identification of habitats where the agent occurs in the area of origin or areas of distribution. These are habitats within which effects may potentially occur in the area of introduction [11]. Data from published literature, data collected during field surveys in the area of origin, and/or data from museum records can be used to determine the type of habitats that the stressor utilizes in the native range or in other areas where they may have been introduced previously. Ecological factors correlated with the relative abundance of the prospective agent can be useful for RA (such as altitude, vegetation type, and other relevant environmental factors).
- Identification of vulnerable habitats in the proposed area of introduction. Once habitats where the agent does occur have been identified, it may be possible to match those habitats with the similar types of habitats present in the area of introduction.
- Identification of habitats that support non-target species. Once non-target species are identified the distribution of these species within the risk region need to be obtained as well as information on temporal occurrence.

2.2. Analysis Phase

This phase involves relating exposure and effects to each other [12]. During this phase of the RA, the exposure of non-target species to the stressor and the magnitude of the effects are characterized.

The assumptions (or risk hypothesis) considered in the analysis phase are the following:

- The sensitivity of non-target species to an introduced BCA varies among habitats [13,14].
- For a non-target species to be at potential risk of non-target effects it must be exposed to the agent spatially and temporally.

2.2.1. Exposure Assessment

Parasitoids will only put native species at risk if they search the habitats of those species and locate and parasitize them in those habitats [14]. Here the potential for spatial overlap of a BC agent with non-target species is described. To be able to overlap spatially the BC agent must successfully disperse to non-target habitats, establish in that habitat and overlap spatially and temporally with the agent.

In the problem formulation phase, potential non-target habitats in the area of introduction are identified based on habitats where the biological control agent is known to occur in other areas, either its place of origin or other areas where the species has been introduced previously. This information provides a baseline prediction of the potential distribution of the BCA in the proposed area of introduction.

Dispersal capacity of the BC agent is an important determinant of the likelihood of exposure of non-target species. Several techniques have been proposed to assess dispersal capacity for augmentative BC agents and could be incorporated in the PRA framework [1,2,9,15]. With the use of GIS, suitable habitats within the dispersal potential of the BC agent could be identified. The probability that agents will leave the release site and find non-target habitats where susceptible non-target species are present can be performed by using techniques such as mark-release-recapture techniques. Results of the dispersal experiments can be used to develop probability distributions for potential spatial overlap. This assessment can be for already in use ABC agents in the area of introduction or also on prospective ABC agents in their respective area or origin or distribution, preferably with similar type of ecosystems.

If ABC agents are likely to establish in either the target habitat or non-target habitats within their dispersal range, RA should be done as if it was an introduction for CBC (other non-target habitats outside its immediate dispersal potential should be considered).

For agents used for CBC the dispersal potential to non-target habitats will be more difficult to determine. Procedures used for ABC are inadequate since these dispersal experiments are usually carried out over a relatively short period time, which will not represent the actual opportunity for long-term dispersal. After establishment, CBC agents (those with both active and passive dispersal) could disperse over time to all potential non-target habitats vulnerable to invasion, unless there are insurmountable geographical barriers that preclude dispersal. In the case of CBC, the distribution of agents in the area of origin and in other areas (e.g. other places where the agent has been released for biological control) can provide useful information on their dispersal potential.

Once potential spatial overlap of non-target species and agent has been analyzed, temporal overlap needs to be assessed. Temporal overlap is based on the time scale of establishment and degree of synchronization with susceptible stages of the non-target hosts [16]. Records of museum collections, published literature as well as survey data can help determine the likelihood of temporal overlap between the BC agent and potential non-target species. It is expected that probability distributions for temporal overlap may be difficult to construct; in such cases, the following scale can be used to account for synchronization with susceptible stages of the non-target host:

0.00 No synchronization with susceptible stages of the non-target host

1.00 Synchronization with susceptible stages of the non-target host.

2.2.2. Effects Assessment

Given exposure, effects on non-target species can occur via direct or indirect mechanisms. Direct effects such as predation or parasitism are readily observed and measured in field and/or laboratory studies, and therefore relatively readily incorporated into risk assessments. Indirect effects are the effects of one species on another mediated by at least one intermediate species such as the case of apparent competition. It has long been recognized that indirect effects could have substantial effects on non-target populations; nevertheless, methods to measure those effects have only recently appeared in the scientific literature [17]. Effects assessment centers on direct effects, since most non-target species selected for PRA in the problem formulation phase are expected to be based on potential direct effects. If the selection is based on potential indirect effects, proposed methods should be followed to quantify these effects [17].

Many studies have shown that data collected during laboratory studies can identify species that are in the agent's physiological host range but not in its ecological host range, and that the former is often greater than the latter [5,18,19]. Also, laboratory studies often cannot predict the actual impact on non-target host populations, which can only be quantified during field studies. Non-target species selected for PRA are considered species with high probability of being used as non-target hosts based on results from laboratory studies. During the effects assessment phase, the magnitude of these non-target effects may be assessed by conducting ecological studies in the agent's area of origin or other areas of distribution. The advantage of conducting this type of study is that the agent and the target host are present, which cannot be done on any realistic scale in the intended place of introduction. Open field experiments in the area of origin of the candidate can provide a more realistic estimation of the magnitude of the non-target use through providing a measure of host specificity under field conditions [20–22] and provide more realistic information and ecological context than information gathered only from quarantine studies. These experiments have the constraint that non-target species that are native to the proposed area of introduction are not present in the agent's area of origin. Surrogate species in the area of origin should then be selected based on phylogenetic and ecological similarities [22]. Results from these studies may provide data that can be used for calculating the percentage of target hosts parasitized in the respective habitat in the area of origin, and to construct probability distributions (describing the probability that a certain percentage predation or parasitism will take place). Experiments of this nature can also provide valuable insights regarding indirect

effects [17]. When field experiments in the area of origin are not feasible, literature records on parasitism rates in different habitats could potentially be used if reliable information is available.

When working with oligophagous or polyphagous species, non-target effects can be influenced by the presence of the preferred or other alternative hosts, which may considerably alter estimates of risk [21,22]. Open field experiments with surrogate species closely related to the target host may also provide insight into how effects may vary in the presence and absence of the preferred or other alternative hosts in different habitats [21]. Field results can be compared with laboratory tests. Life table studies on surrogate species closely related to the non-target hosts may also provide useful information on the relative impact of the agents on population of non-target species. Life table studies on non-target hosts in the country of proposed introduction can also provide useful information regarding the extent to which current mortality factors (e.g. existing natural enemies) affect the population of the non-target species, and estimate the level of effect that can be posed if the agent is released [7].

Collaboration with universities and research centers in the area of origin of the proposed candidate can provide a means to conduct such studies.

2.3. Risk Characterization

This phase of the RA involves fitting data into the precision tree. The estimation of risk requires the integration of exposure and effects data and the evaluation of any associated uncertainties [12].

In this phase, probabilistic risk assessment is implemented by constructing precision trees. Figure 1 shows a conceptual decision tree that incorporates aspects such as potential to disperse to suitable habitats in the area of release, potential to overlap temporally with non-target species, and potential to cause direct or indirect effects on non-target species. The conceptual decision tree can incorporate some other variables that may be important in determining the risk in specific cases, whereas other components can be excluded if not important for the specific case. In this conceptual tree, the probability to disperse to suitable habitats is a fundamentally important factor determining the risk of exposure to non-target species, also spatial and temporal overlap with non-target species are of major interest in characterizing the risk. The 'overlap with non-target species' node can be further divided into multiple branches for as many non-target species that are be included in the assessment.

The probability that the agent will cause an effect on non-target populations (and therefore be part of its ecological host range) will be conditional on a habitat overlap of non-target species with the biological control agent that in turn, is conditional on the successful dispersal of the agent to non-target habitats. Data collected in the analysis phase are used to fit the precision tree in the sequence of events [9]. Probabilities are multiplied along branches to calculate the overall P-value that the BC agent will attack a non-target species. The multiplication of the overall P-value (as an estimate of proportion of the population likely to be impacted) by the density of the non-target species might provide an estimate of the extent of that impact on a population of the non-target species. This is an aspect that requires additional attention, as there are numerous factors that need to be considered, such as seasonal and geographic variability in non-target population densities.

When PRA is conducted on a preferred or target host in a non-target habitat, the results will only indicate that the BC agent is present and able to forage for the target hosts but cannot be used to infer the magnitude of effects on a non-target host. However, if the effects assessment part is carried out with non-target hosts or surrogate species that are ecological and phylogenetically close to the non-target species, in a similar habitat then results of the risk assessment will approximate the probability that the agent will actually parasitize the non-target host in a specific type of habitat.

Uncertainty Analysis

In this step the uncertainty in the prediction resulting from the uncertain data used is assessed. Data used in any risk assessment are subject to uncertainties from different sources. Therefore, the decision maker needs to understand and quantify the uncertainties associated with the scientific information on which the decision will be based [8]. Sources of uncertainty in risk assessment may

arise from (1) inherent variability of the data (stochasticity); (2) data gaps; and (3) mistakes in the assessment activities (investigator error). Uncertainty originating from data gaps and investigator error can be reduced but uncertainty originating from variability of the data can only be described and estimated, but not reduced [8].

Uncertainty is modeled by using Monte Carlo uncertainty analysis. Uncertainty analyses (Monte Carlo simulations) can be performed with computer software such as Crystal Ball [23] or @Risk (Palisade Corporation 2002) [24]. Monte Carlo (MC) analysis is a type of probabilistic approach used to quantify the change in model outputs as a function based on probability distributions of each of the uncertain parameters of the model. The basic steps for Monte Carlo simulation analysis are the following [8]: (1) defining the statistical distributions of the input parameters, (2) randomly sampling from these distributions, (3) performing repeated model simulations using the randomly selected set of parameters, and (4) analyzing the output. The outputs of the analysis are presented in a form of statistical distribution (uncertainty distribution) representing the entire range of possible outcomes (e.g. impact on a non-target species) and the likelihood of each outcome being realized.

Uncertainty analysis provides the basis for efficient data collection, and application of refined methods [12] or justifies a degree of conservatism in the predicted outcomes in the face of uncertainty [8]. Uncertainty analysis can therefore increase the confidence in a decision.

References

1. Van Lenteren, J.C.; Babendreier, D.; Bigler, F.; Burgio, G.; Hokkanen, H.T.M.; Kiske, S.; Loomans, .J.M.; Menzler-Hokkanen, I.; Van Rijn, P.C.J.; Thomas, M.B.; et al. Environmental risk assessment of exotic natural enemies used in inundative biological control. *BioControl* **2003**, *48*, 3–38.
2. Van Lenteren, J.C.; Bale, J.; Bigler, F.; Hokkanen, H.M.T.; Loomans, A.J.M. Assessing risks of releasing exotic biological control agents of arthropod pests. *Ann. Rev. Entomol* **2006**, *51*, 609–634.
3. Van Lenteren J.C.; Loomans A.JM. Environmental risk assessment: Methods for comprehensive evaluation and quick scan. In *Environmental Impact of Invertebrates for Biological Control of Arthropods: Methods and Risk Assessment*; Bigler, E., Babendreier, D., Kuhlmann, U., Eds.; CABI Publishing: Wallingford, Oxon, UK, 2006; pp. 254–272.
4. Barron M.C.; Wratten, S.D.; Barlow, N.D. Non-target parasitism of the endemic New Zealand red admiral butterfly (Bassaris gonerilla) by the introduced biological control agent *Pteromalus puparium*. *Biol. Control* 2003, 27, 329–335.
5. Benson, J.; Pasquale, A.; Van Driesche, R.; Elkinton, J. Assessment of risk posed by introduced braconid wasps to *Pieris virginiventris*, a native woodland butterfly in New England. *Biol. Control* **2003**, *26*, 83–93.
6. Haye, T., Goulet, H., Mason, P.G., Kuhlmann, U. Does fundamental host range match ecological host range? A retrospective case study of a *Lygus* plant bug parasitoid. *Biol. Control* **2005**, *35*, 55–67.
7. Johnson, M.T.; Follett, P.A.; Taylor, A.D.; Jones, V.P. Impacts of biological control and invasive species on a non-target native Hawaiian insect. *Oecologia* **2005**, *142*, 529–540
8. Suter, G.W., II. *Ecological Risk Assessment*; Lewis Publishers: Boca Raton, FL, USA, 1993; p. 538.
9. Wright, M.G.; Hoffmann, M.P.; Kuhar, T.P.; Gardner, J.; Pitcher, S.A. Evaluating risks of biological control introductions: A probabilistic risk-assessment approach. *Biol. Control* **2005**, *35*, 338–347.
10. Bier, V.M. An Overview of Probabilistic Risk Analysis for Complex Engineered Systems. In *Fundamentals of Risk Analysis and Risk Management;* Molak, V., Ed.; CRC Press: Boca Raton, FL, USA, 1997.
11. USEPA (U.S. Environmental Protection Agency). *Framework for ecological risk assessment.* Washington, DC: Risk Assessment Forum, U.S. Environmental Protection Agency. EPA/630/R-92/001. 1992.
12. USEPA (U.S. Environmental Protection Agency). Guidelines for Ecological Risk Assessment. U.S. Environmental Protection Agency, Risk Assessment Forum, Washington, DC, EPA/630/R095/002F. 1998.
13. Wiegers, J.K.; Feder, H.M.; Mortesen, L.S.; Shaw, D.G.; Wilson, V.J.; Landis, W.G. A regional multiple stressor rank-based ecological risk assessment for the Fjord of Port Valdez. *Hum. Ecol. Risk Assess.* **1998**, *4*, 1125–1173.
14. Colnar, A.M.; Landis, W.G. Conceptual model development for invasive species and a regional risk assessment case study: The European green crab, *Carcinus maenas*, at Cherry Point, Washington, USA. *Hum. Ecol. Risk Assess.* **2007**, *13*, 120–155.

15. Sands, D.P.A.; Van Driesche, R.G. Using the Scientific literature to estimate the host range of a biological control agent. In *Assessing Host Ranges for Parasitoids and Predators Used for Classical Biological Control: A Guide to Best Practice*; Van Driesche, R.G., Reardon, R., Eds.; Department of Agriculture Forest Health Technology Enterprise Team: Morgantown, WV, USA, 2004; pp. 15–23.
16. Van Lenteren J.C.; Loomans A.JM. Environmental risk assessment: Methods for comprehensive evaluation and quick scan. In *Environmental Impact of Invertebrates for Biological Control of Arthropods: Methods and Risk Assessment*; Bigler, E., Babendreier, D., Kuhlmann, U., Eds.; CABI Publishing: Wallingford, Oxon, UK, 2006; pp. 254–272.
17. Stiling, P. Why do natural enemies fail in biological control campaigns? *Am. Entomol.* **1993**, *39*, 31–37.
18. Messing, R.H.; Roitberg, B.; Brodeur, J. Measuring and predicting indirect impacts of biological control: competition, displacement and secondary interactions. In *Environmental Impact of Invertebrates for Biological Control of Arthropods: Methods and Risk Assessment*; Bigler, E., Babendreier, D., Kuhlmann, U., Eds.; CABI Publishing: Wallingford, Oxon, UK, 2006; pp. 64–77.
19. Onstad, D.W.; McManus, M.L. Risks of host range expansion by parasites of insects. *BioScience* **1996**, *46*, 430–435.
20. Cameron, P.J. Walker, G.P. Host specificity of *Cotesia rubecula* and *Cotesia plutellae*, parasitoids of white butterfly and diamondback moth. In *Proceedings of the 50th New Zealand Plant Protection Conference*. 1997, pp. 236–241.
21. Briese, D.T.; Zapater, M.; Adorno, A.; Perez-Camargo, G. A two-phase open-field test to evaluate host specificity of candidate biological control agents for *Heliotropium amplexicaule*. *Biol. Control* **2002**, *25*, 273–287.
22. Briese, D.T. Translating host-specificity test results into the real-world: The need to harmonize the yin and yan of current testing procedures. *Biol. Control* **2005**, *35*, 208–214.
23. Decisionneering. Crystal Ball Risk Analysis Software. Available online: www.decisioneering.com (accessed on-9 January 2005).
24. Palisade Corporation. *@ Risk advanced risk analysis for spreadsheets*. Palisade Corporation: Newfield, NY., USA, 2002.

© 2017 by the authors. Submitted for possible open access publication under the terms and conditions of the Creative Commons Attribution (CC BY) license (http://creativecommons.org/licenses/by/4.0/).
